# Whole-Genome Sequencing and Comparative Genome Analysis of Fusarium solani-melongenae Causing Fusarium Root and Stem Rot in Sweetpotatoes

**DOI:** 10.1128/spectrum.00683-22

**Published:** 2022-07-07

**Authors:** Shu-Yan Xie, Tingting Ma, Nan Zhao, Xinxin Zhang, Boping Fang, Lifei Huang

**Affiliations:** a Crops Research Institute, Guangdong Academy of Agricultural Sciences, Guangdong Provincial Key Laboratory of Crop Genetic Improvement, Guangzhou, China; b College of Plant Protection, South China Agricultural University, Guangzhou, China; c College of Agriculture and Biology, Zhongkai University of Agriculture and Engineering, Guangzhou, China; University of Molise

**Keywords:** *Fusarium solani-melongenae*, *Fusarium solani* species complex, genome sequencing, comparative genome analysis, Fusarium root and stem rot, sweetpotato

## Abstract

Sweetpotato (Ipomoea batatas) is the eighth most important crop globally. However, the production and quality of sweetpotatoes are threatened by Fusarium diseases that are prevalent around the world. In this study, a Fusarium species that causes root and stem rot in sweetpotatoes was studied. The pathogenic fungus CRI 24-3 was isolated and sequenced using third- and next-generation sequencing techniques and a 49.6 Mb chromosome-level draft genome containing 15,374 putative coding genes were obtained. Molecular phylogenetic analysis showed that CRI 24-3 was an F. solani-melongenae strain within clade 3 of the F. solani species complex (FSSC). CRI 24-3 showed a relatively high number of virulence factors, such as carbohydrate-active enzymes (CAZymes), pathogen-host interaction (PHI) proteins, and terpene synthases (TSs), compared with the number of those identified in other sequenced FSSC members. Comparative genome analysis revealed considerable conservation and unique characteristics between CRI 24-3 and other FSSC species. In conclusion, the findings in the current study provide important genetic information about F. solani-melongenae and should be useful in the exploration of pathogenicity mechanisms and the development of Fusarium disease management strategies.

**IMPORTANCE** Fusarium root and stem rot in sweetpotato are prevalent in the main sweetpotato-growing areas in China, and fungal isolation, morphological characteristics, and molecular phylogenetic analysis of the disease causal agent (F. solani-melongenae isolate CRI 24-3) were systematically studied. The genome sequence of F. solani-melongenae isolates CRI 24-3 was first reported, which should provide a basis for genome assembly of other closely related Fusarium species. Carbohydrate-active enzymes predicted in CRI 24-3 may be important to convert the substantial polysaccharides to sustainable and renewable energy. Moreover, other virulence factors facilitating Fusarium diseases, including effectors and toxic secondary metabolites, are ideal objects for pathogenicity mechanism research and molecular targets for fungicide development. The findings of comparative genome analysis of CRI 24-3 and 15 sequenced members of the F. solani species complex help promote an integral understanding of genomic features and evolutionary relationships in Fusarium.

## INTRODUCTION

Sweetpotato (Ipomoea batatas) is a high-yield crop plant cultivated around the world. It ranked eighth in global annual production in 2020 as reported by the Food and Agriculture Organization (https://www.fao.org/home/en). Sweetpotato is an important food source for humans and other animals and serves as pharmaceutical and chemical raw materials. However, the production and quality of sweetpotatoes are significantly limited by Fusarium diseases (1). The genus Fusarium (Nectriaceae, Hypocreales) comprises at least 450 phylogenetic species within 23 divergent species complexes (2, 3). The Fusarium solani species complex (FSSC) is one of the most important lineages in Fusarium and is notorious for causing diseases in plants, humans, and other animals (4). The FSSC encompasses more than 100 species divided into three clades (clades 1 to 3), of which clade 3 is the largest and most of the pathogenic species (5). Moreover, a few species in clade 3 produce perithecial sexual morphs described in *Nectria*, *Neocosmospora*, *Haematonectria,* and *Hypomyces* (6, 7). However, following the demise of dual nomenclature on 1 January 2013, species in the FSSC should be reported as Fusarium (2, 5).

Phenotypic characterization is fundamental and critical for Fusarium species classification and identification (6). However, Fusarium species share similar morphological characteristics and change rapidly in response to the external environment. Therefore, it is challenging to accurately classify these fusaria based only on morphological traits. Sequence-based molecular analysis is currently a powerful tool for comprehensive fungal studies (8, 9). The internal transcribed spacer (ITS) region is a DNA marker widely used for fungal taxonomy due to its high conservation within members of the same genus, interspecific variation, and high availability (8, 10). Other gene markers such as RNA polymerase II second largest subunit (*RPB2*) and translation elongation factor 1-α (*TEF 1-α*) are also used to explore fungi at multiple taxonomic levels (9, 11). Moreover, omics techniques such as genome or transcriptome sequencing, have significantly improved our understanding of taxonomy, genome evolution, genetic diversity, and pathogenic mechanisms (12–15). Currently, only 16 sequenced members out of over 100 FSSC species have been publicly available in the National Center for Biotechnology Information (NCBI) database. The genome of F. vanettenii 77-13-4 (formerly referred to as Nectria haematococca MPVI or F. solani f. sp. pisi) provided the first genomic insight into this large species complex (12). Interaction between F. vanettenii and its hosts represents a key model system for plant-fusaria studies (16). Comparative genome analyses of F. vanettenii and other sequenced fusaria have revealed two distinct functional compartmentalization of Fusarium genomes with significantly different genome sizes due to evolutionary genetic events (13, 15–17).

The plant cell wall is a natural barrier characterized by high mechanical strength, and it mainly comprises celluloses, hemicelluloses, and pectins, which play significant roles in protecting plants from fungal infection (18, 19). Secreted carbohydrate-active enzymes (CAZymes) can decompose plant cell walls by catalyzing hydrolyzation, modification, and formation of glycosidic bonds. Genome-wide identification and comparison of genes encoding CAZymes in fungi showed their roles in causing diseases and the potential of producing renewable biofuels and biochemicals (20–23). Furthermore, toxic secondary metabolites produced by fusaria are responsible for plant diseases and food contamination in humans and livestock. Genome comparison analyses of Fusarium species have revealed a tandem array of genes encoding secondary metabolites, including polyketide synthase (*PKS*), nonribosomal peptide synthetase (*NRPS*), and terpene synthase (*TS*), which are involved in the catalysis of key fungal secondary metabolites (17). Mating type (*MAT*) locus, including *MAT1-1* and *MAT1-2* idiomorphs, regulates *PKS* gene expression and is critical for ascospores production in the sexual cycle in Fusarium species, and the underlying mechanisms have been studied (17, 24). Normally, the *MAT* locus was flanked by *AP endonuclease 2* (APN2) and *Synthetic Lethal with ABP1* (SLA2) genes. Both *MAT1-1* and *MAT1-2* idiomorph genes are present at the *MAT* locus in the homothallic species that are capable of selfing, whereas the heterothallic Fusarium species contained either *MAT1-1* or *MAT1-2* idiomorph genes, which are self-sterile and failed to produce selfed perithecium without the opposite mating type. It seems that the *MAT1-1* and *MAT1-2* idiomorph genes develop a complementary relationship in the facilitation of mating compatibility.

Before being formally described, F. solani-melongenae was informally referred to as FSSC 21 within clade 3. This species infects a wide range of agronomically important plants, inducing root rot, stem wilting, and blight (25, 26). It has undergone several nomenclatural changes, including a *formae speciales* of F. solani (6, 7, 26, 27). However, F. solani-melongenae is morphologically and evolutionarily distinct from F. solani (3, 23, 28). During the past few years, a severe Fusarium disease causing yield loss and quality deterioration of sweetpotatoes, named Fusarium root and stem rot (FRST) by Huang et al. (1), has spread in several main sweetpotato-growing provinces in China, including Guangdong, Zhejiang, Fujian, and Hainan. FRST has gradually become a major sweetpotato disease, but the pathogen remained unidentified. In the current study, the FRST pathogen was isolated and identified as F. solani-melongenae by morphological analysis, genome sequencing, and molecular phylogenetic analyses. Furthermore, analyses of virulence factors and genome comparison between F. solani-melongenae and other fusaria were conducted. To the best of our knowledge, this was the first detailed study of the F. solani-melongenae genome and comparative analysis of 15 other sequenced FSSC genomes. This study provides key findings on the characteristics of F. solani-melongenae and may be a basis for the development of strategies to prevent and control FRST disease.

## RESULTS

### Fungal isolation and morphological analysis revealed that CRI 24-3 was the pathogen of FRST.

**FIG 1:**
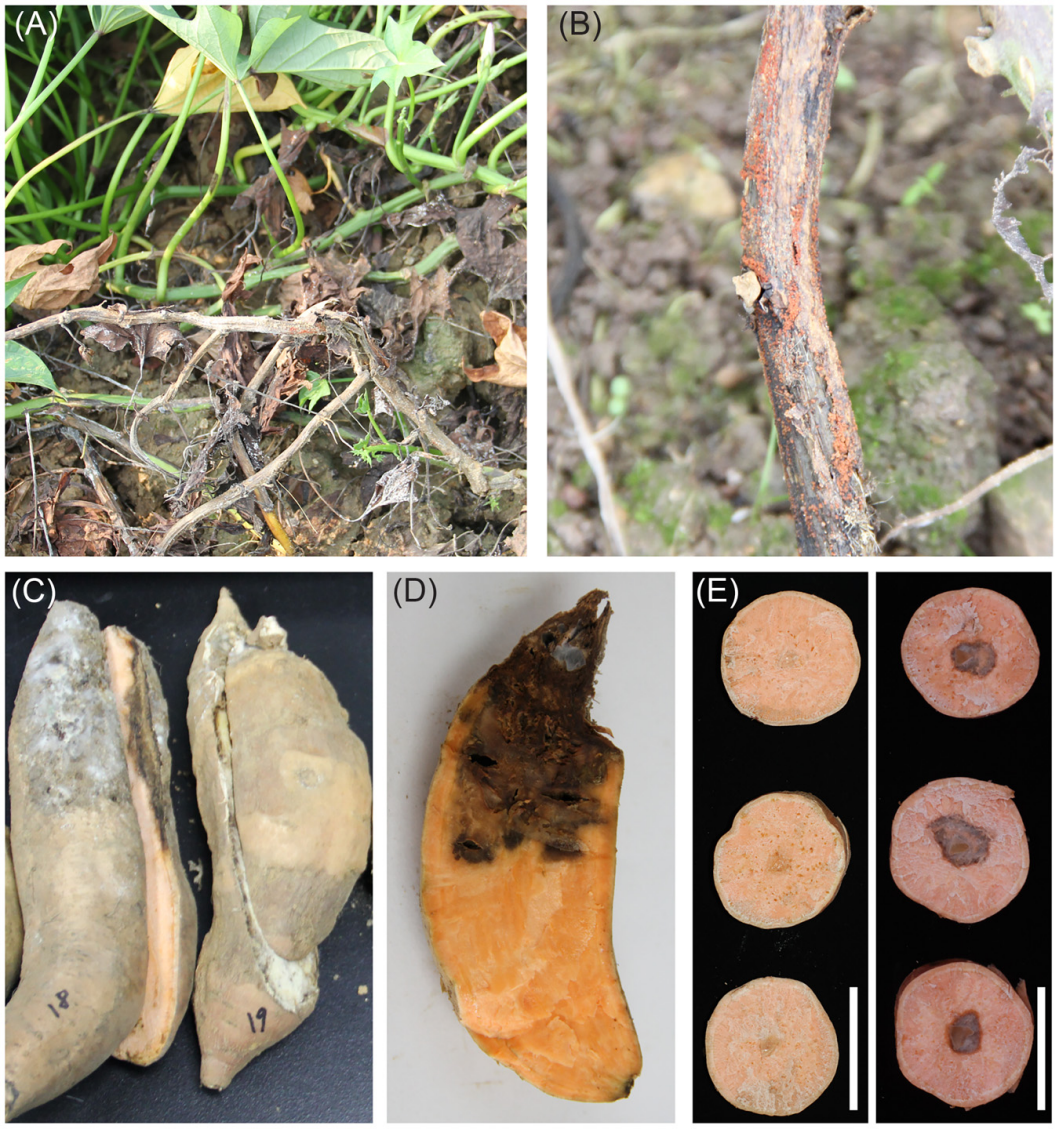
Phenotypes of FRST-infected sweetpotato plants in the field. (A) Infected stems and leaves. (B) Reddish perithecia covering the basal stem. (C and D) Infected storage roots. (E) Negative-control (left) and storage roots inoculated with CRI 24-3 (right) in pathogenicity test. Bar = 5 cm.

Field analysis indicated that water-soaked lesions initially appeared on the basal stem of the FRST-infected sweetpotato (Fig. 1A). Moreover, abundant reddish perithecia superficially covering the necrotic basal stem were observed during humid or rainy weather (Fig. 1B). The upper stem and leaves gradually became chlorotic and blighted as the disease spread. Storage roots exhibited concave lesions with clear cyclic margins, and white hyphae were distributed over sunken lesions together with cracked necrotic tissues inside the tuber (Fig. 1C and D), thus significantly decreasing sweetpotato yield and quality. A dominant fungal colony CRI 24-3 was isolated from the diseased sweetpotato samples. A pathogenicity test of CRI 24-3 was then conducted based on Koch’s postulates. Watery-brown lesions with sparse hyphae extending past the inoculation point were observed on the sliced roots, which were absent in the negative-control (Fig. 1C), indicating that CRI 24-3 was the causal agent of FRST in sweetpotato.

**FIG 2:**
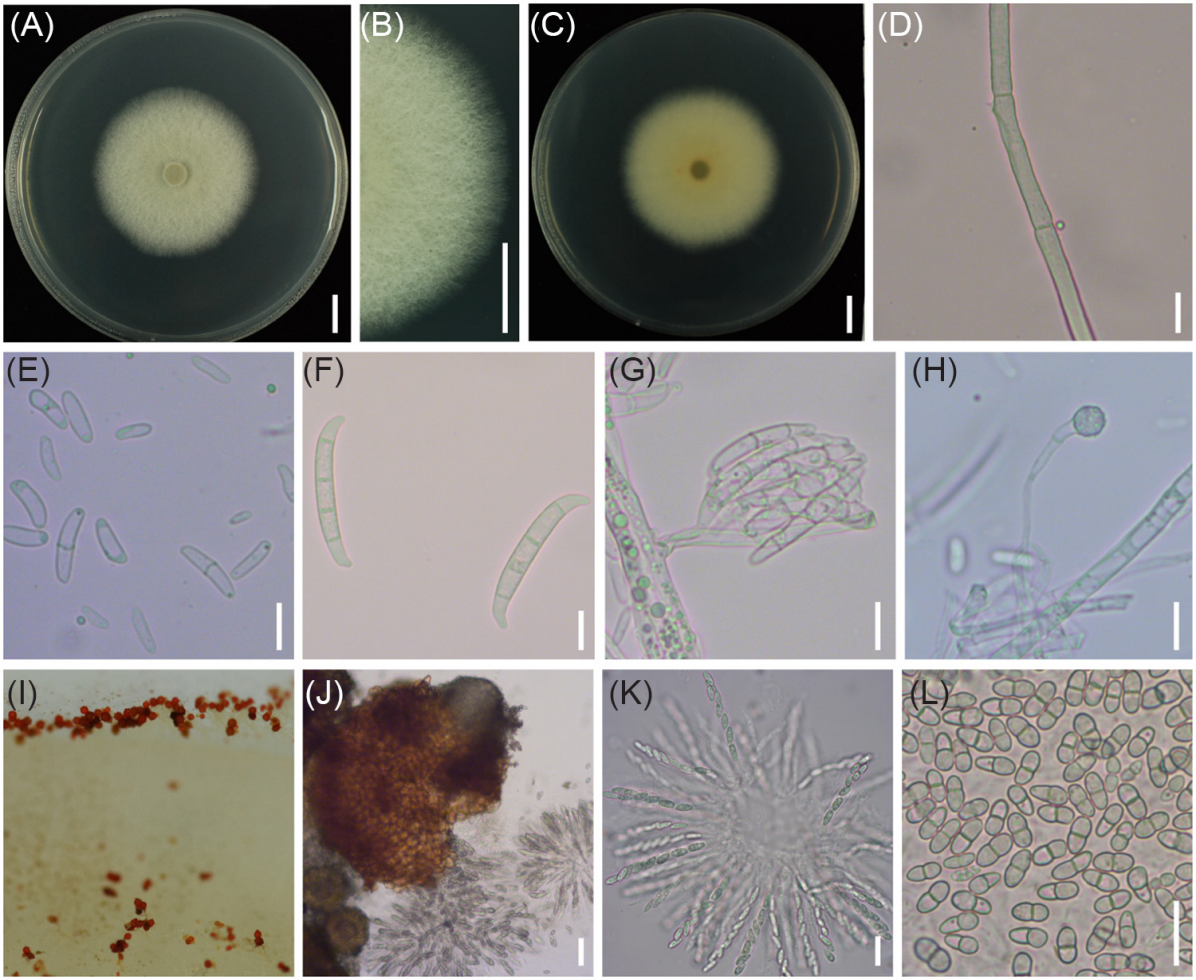
Morphological characteristics of CRI 24-3 on PSA. (A to C) Colony from the surface and reverse after culture for 4 days. Bar = 1 cm. (D) Aerial hyphae. (E) Microconidia. (F) Macroconidia. (G) Conidia in the false head. (H) Chlamydospore. Bar = 10 μm. (I) Perithecia. (J) Enlarged perithecium. Bar = 50 μm. (K) Ascus bearing (L) Eight ascospores. Bar = 20 μm.

CRI 24-3 was cultured on potato sucrose agar (PSA) and synthetic low nutrient agar (SNA) for further morphological analysis. The mean colony diameter on PDA was 4.4 cm after culture in an incubator (25°C, 12 h photoperiod, and 75% relative humidity) for 4 days. The fluffy colony had septate aerial mycelia that spread over the plate. The colony was creamy-white on the surface and yellow on the reverse (Fig. 2A to C). Microconidia were abundant, ellipsoidal to reniform, and most were 1-septate (Fig. 2E). The 1-septate microconidia were (7.8 to 13.8) × (2.1 to 3.9) μm. Macroconidia were falcate, 3- to 5-septate, and had slightly curved apical cells and foot-like basal cells (Fig. 2F). The average width of the 3- to 5-septate macroconidia was 4.1 to 4.9 μm. Terminal or intercalary chlamydospores (6.1 to 7.4 μm) from short lateral branches were spherical and coarse (Fig. 2H). After 4 weeks, the colony was covered with dotted dark red perithecia, similar to those on the infected sweetpotatoes in the field (Fig. 1B and 2I). The pear-shaped perithecium of CRI 24-3 had a papillate neck and ostiole (Fig. 2J) through which the ascospores were released. Moreover, the clavate asci contained eight uniseriate 1-septate ascospores that were (10.7 to 12.6) × (3.9 to 5.0) μm in size (Fig. 2K and L). In contrast, the colony on SNA exhibited weaker growth (Fig. S1) with sparse substrate hyphae and a mean diameter of 4.1 cm after 4 days. The number of conidia and perithecia of CRI 24-3 on SNA was less relative to that on PSA. However, the morphological characteristics of the conidia and perithecia were similar to those observed on PDA. A representative culture of isolate CRI 24-3 (accession no. ACCC 39784) was deposited in the Agricultural Culture Collection of China (http://www.accc.org.cn/).

### A high-quality draft genome assembly of CRI 24-3 was generated and annotated.

**FIG 3:**
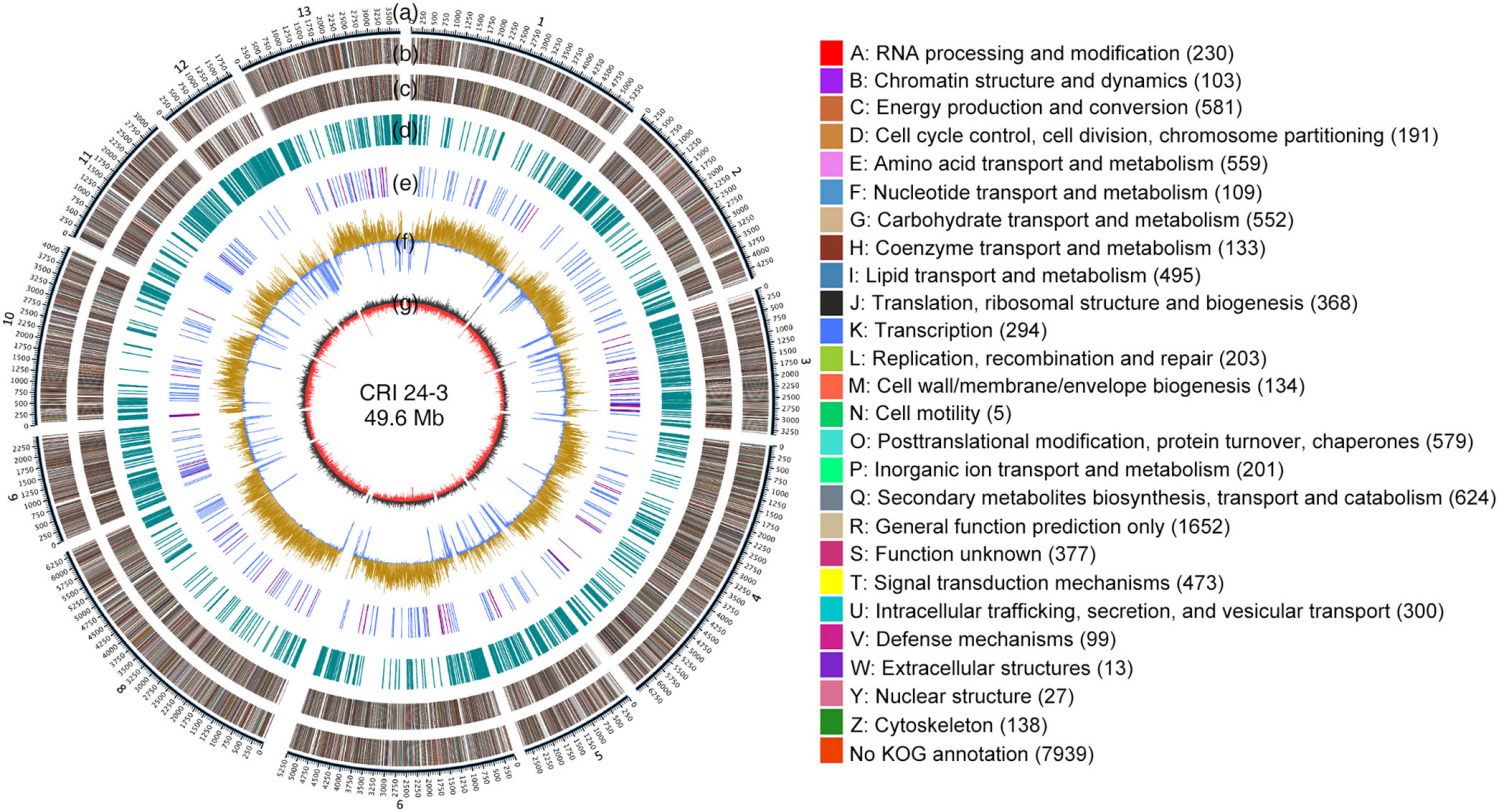
Circular map of CRI 24-3 genome assembly. (A) The physical location of 12 contigs. Bar = 1 kb. (B and C) Protein-coding genes on the forward and reverse strand with KOG classes. (D) Repetitive sequences. (E) tRNA (blue) and rRNA (purple) coding genes on the forward strand. (F) GC content in certain regions was higher (yellow) or lower (blue) than the average genome GC content. (G) GC-skew = (G−C)/(G+C) with positive (black) or negative (red) value.

A total of 452,011 high-quality clean reads (average length 13,525.1 bp) were obtained after quality control (Table S1). A 49.6 Mb *de novo* chromosome-level genome of CRI 24-3 was generated from 12 contigs with a 4.5 Mb Contig N50 and 50.7% GC content under an estimated 123.3-fold coverage depth (Fig. 3; Table 1). Results of genome quality assessment indicated that the genome assembly of CRI 24-3 was complete and accurate as single-copy orthologous genes in CRI 24-3 matched 99.9% of all 3,817 complete core genes in the sordariomycetes_odb10 data set (Table 1).

**TABLE 1:** Features of CRI 24-3 genome assembly

Genome information	
Genome size (bp)	49,564,288
Repeated sequences (bp)	2,348,420
Coverage (%)	99.7
Depth of coverage	123.3×
No. of scaffolds	12
Contig length (bp)	49,564,288
Contigs N50 (bp)	4,496,268
No. of putative genes	15,374
Average gene length (bp)	2,019.3
GC content (%)	50.7
Complete BUSCOs	3,813
Fragmented BUSCOs	0
Missing BUSCOs	4
No. of tRNA	313
No. of rRNA	89

A total of 15,374 putative genes with an average gene length of 2.0 kb were identified using three gene structure constructing methods (Fig. S2A; Table 1). Moreover, 15,256 protein-coding genes were annotated through multiple functional databases based on sequence similarity comparison (Table 2). About 80.3% of the protein-coding genes in CRI 24-3 matched the genome of F. vanettenii 77-13-4 based on the Non-Redundant Protein Sequence (Nr) database analysis (Fig. S2B), which indicated these two species closely related. Furthermore, 10,655 protein-coding genes were subjected to Gene Ontology (GO) analysis (Table 2) and were functionally assigned to three classes: “cellular component” (7,100), “molecular function” (8,279), and “biological process” (8,069). The “catalytic activity” (5,673), “metabolic process” (5,452), and “binding” (4,353) were the top three most enriched terms (Fig. S3). Further functional analysis using the euKaryotic Orthologous Groups (KOG) database showed that the “general function prediction only” category (R, 22.2%) had the highest number of proteins, followed by the “secondary metabolites biosynthesis, transport, and catabolism” category (Q, 8.4%) and “energy production and conversion” category (C, 7.8%) (Fig. 3). Moreover, an analysis of 3,925 coding genes using the Kyoto Encyclopedia of Genes and Genomes (KEGG) database showed that 2,329 genes were enriched in 114 pathways belonging to five categories (Fig. S4; Table 2). Most genes were enriched in pathways of carbohydrate (471) and amino acid metabolism (435).

**TABLE 2:** All databases used for CRI 24-3 genome annotation

Database	No.	100≤length<300	Length ≥300
Nr	15,252	3,822	11,180
GO	10,655	2,209	8,315
KOG	7,435	1,392	5,985
KEGG	3,925	900	2,957
Pfam	11,519	2,355	9,074
Swiss-Prot	9,092	1,666	7,337
TrEMBL	15,252	3,821	11,180
All	15,256	3,825	11,180

### CRI 24-3 was identified as F. solani-melongenae based on molecular phylogenetic analysis.

**FIG 4:**
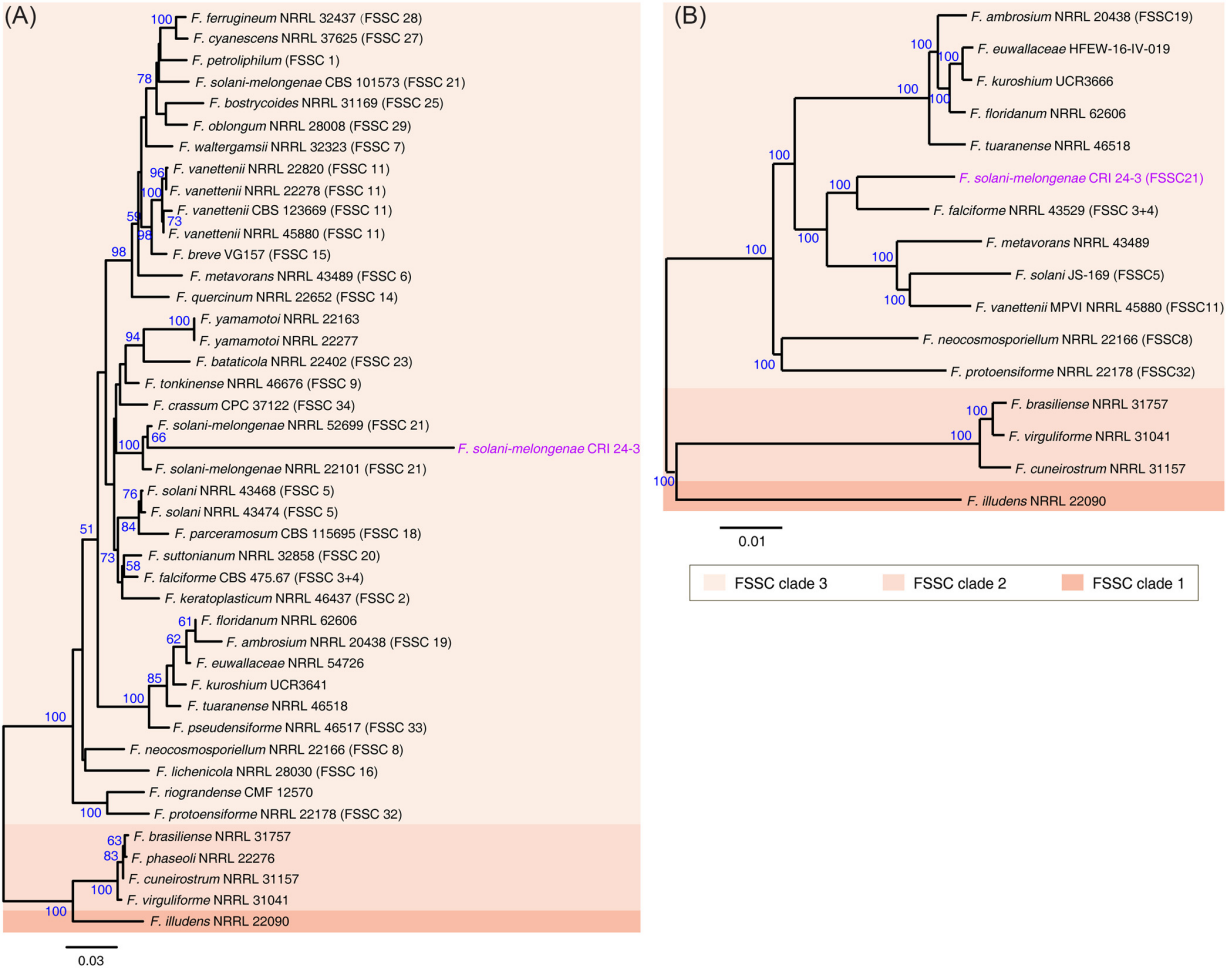
Phylogeny analysis of CRI 24-3 and other phytopathogenic Fusarium species. ML tree inferred from (A) concatenate sequences of ITS, *RPB2*, and *TEF 1-α*, and (B) combined sequences of 1,544 single-copy homologous genes shared by 16 FSSC species. Bootstrap values (blue) indicate the percentage of trees where the associated strain clustered together. Arabic numerals in brackets distinguish the phylogenetically diverse species within FSSC clade 3.

An alignment against the NCBI database using the BLASTn tool indicated that the ITS sequence PCR amplified from CRI 24-3 genomic DNA shared 99.8% identity with that of F. solani-melongenae NRRL 22101 with 100% query coverage (unpublished data), indicating CRI 24-3 is a member of the FSSC. To identify CRI 24-3 more accurately, an informative phylogenetic analysis based on the concatenated sequences of *ITS*, *RPB2*, and *TEF 1-α* was performed (Fig. 4A). Most bootstrap values in the combined sequences tree were higher than those in the ITS tree (unpublished data). The three-loci tree showed a dominant branch comprising 38 ingroup terminals within the FSSC clade 3, to which taxa within the FSSC clades 1 and 2 formed a basal sister group (Fig. 4A and Table S2). The major branch formed by the FSSC taxa was split into one terminal node, including CRI 24-3 and another sub-basal node comprising F. protoensiforme (FSSC 32) and F. riograndense. Above all, CRI 24-3 was closely related to F. solani-melongenae and clustered within FSSC clade 3. Therefore, the isolate CRI 24-3 that caused FRST was identified as F. solani-melongenae (FSSC 21) based on the method proposed by Nilsson et al. (10).

The phylogenomic relationship between CRI 24-3 and 15 genomically sequenced FSSC species was explored (Table S3). F. illudens NRRL 22090 was used as an outgroup to generate a rooted phylogenomic tree. The whole-genome phylogram from the 1,544 single-copy homologous genes shared by 16 fusaria genomes was well-supported, in which 14 nodes had 100% bootstrap values (Fig. 4B). The 16 FSSC species were divided into three known clades. Phylogenomic relationship analysis showed CRI 24-3 was closely related to F. falciforme (FSSC 3 + 4) with 100% bootstrap support. The two species formed a monophyletic group with F. vanettenii, F. solani, and F. metavorans, indicating that these species may share an ancestor.

### Abundant virulence factors included unique trichodiene synthases were found in CRI 24-3.

**FIG 5:**
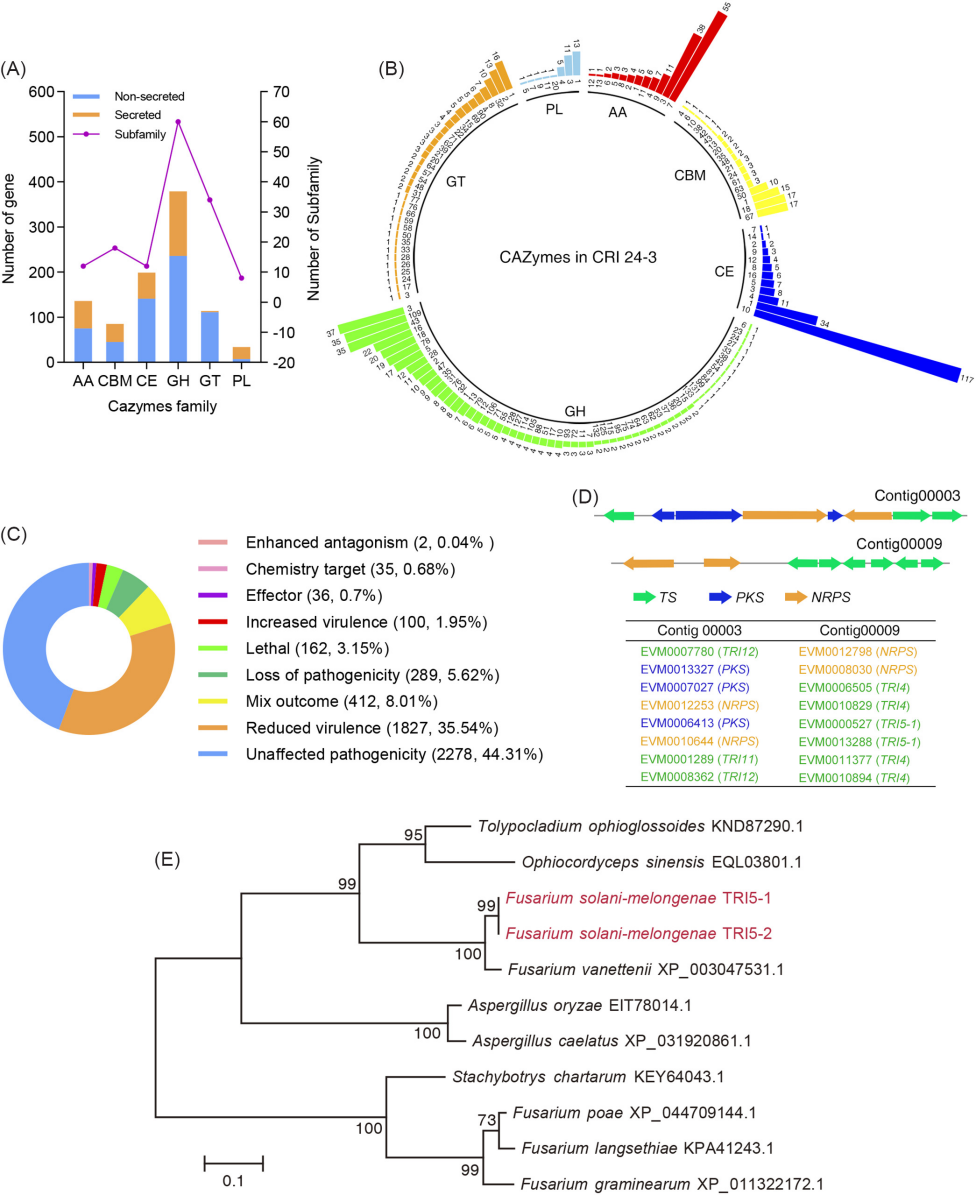
CAZymes and PHI proteins in CRI 24-3 proteome. (A) Number of CAZymes and their relevant family. (B) CAZymes family distribution. Numbers near the internal circle and at the top of the bars represent the family ID and the number of the proteins, respectively. AA: auxiliary activity, CBM: carbohydrate-binding module, CE: carbohydrate esterase, GH: glycoside hydrolase, GT: glycosyl transferase, PL: polysaccharide lyase. (C) Functions of 5141 PHI proteins. The mixed outcome indicates that at least two distinctive functions were observed in one protein. (D) Tandem array genes encoding PKS, NRPS, and TS clustered in Contig00003 and Contig00009 of CRI 24-3 assembly. (E) Phylogenetic relationships analysis inferred from 11 trichodene synthase sequences from CRI 24-3 and other related species.

In this study, 889 putative CAZymes-coding genes were identified in CRI 24-3 (Table S4), which may be implicated in the prominent carbohydrate metabolic pathway as described previously (Fig. 3; Fig. S4). The number of CAZymes in CRI 24-3 was higher relative to that in some other fusaria (e.g., 629 in F. virguliforme within the FSSC clade 2; 551 in F. neocosmosporiellum within the FSSC clade 3; and 481 in F. graminearum from the F. sambucinum species complex). The CAZymes were grouped into six classes, which differ in family distribution and gene number (Fig. 5A). Glycoside hydrolases (GHs, 379) were the biggest class, followed by carbohydrate esterases (CEs, 199), and auxiliary activities (AAs, 136). CRI 24-3 had 60 GH families with most enzymes distributed in GH3 (37), GH43 (35), GH109 (35), whereas some common GH subfamilies, such as GH29, GH30, and GH44, were not present in CRI 24-3 (Fig. 5B), similar to the CAZyme profile in F. virguliforme (19). Three hypothetical β-1,4-xylanases from GH11 referred to as FsmGH11.1, FsmGH11.2, and FsmGH11.3 were identified in CRI 24-3 (Table S4). The multiple alignments (Fig. S5) showed that FsmGH11.3 had the highest sequence similarity (44.1%) with NhGH11 (GenBank accession no. XP_003050975.1), a β-1,4-xylanase from GH11 in F. vanettenii was proven to decompose various xylan substrates (29). This implied that FsmGH11.3 and NhGH11 may share similar enzymatic hydrolysis of xylan.

More virulence and effector genes influencing pathogen-host interactions were further evaluated. A total of 5,141 PHI coding genes (33.7% of CRI 24-3 genome) including 36 effectors and 100 virulence factors were identified (Fig. 5C; Table S5). Moreover, 1,154 secreted proteins were predicted, which were even more compared with those reported in F. oxysporum (12), the species with the largest known genome in Fusarium (Table 3). In addition, 180 hypothetical effectors secreted with signal peptides without a transmembrane region were identified (Table S6), which may confer pathogenicity and are thus potential targets for gene editing. In the current study, products of two genes (EVM0000527 and EVM0013288) were functionally annotated as trichodiene synthase (named TRI5-1 and TRI5-2, respectively) through the Pfam and Swissprot databases, with 44.7% identity under a relatively low E value (1.2645E-118). Additionally, 13 trichodiene oxygenases (TRI4), and four trichothecene efflux pumps (TRI12) involved in the terpene-producing cascade reaction, together with 17 PKSs and 12 NRPSs, were also identified in the CRI 24-3 proteome, among which only one PKS (EVM0010851.1) was predicted as an effector (PHI: 325) in CRI 24-3 (Table S5 and S6). Notably, two major gene clusters in Contig00003 and Contig00009 that encode synthases described above were observed in the CRI 24-3 assembly (Fig. 5D). Phylogenetic relationship analysis of 11 trichodiene synthases from CRI 24-3, Fusarium species, and related species in other genera showed TRI5-1 and TRI5-2 from CRI 24-3 were closely related to an uncharacterized protein (XP_003047531.1) from F. vanettenii, grouped tightly with other homologous trichodiene synthases from divergent genus rather than from Fusarium species (Fig. 5E). Conserved protein domain searches in the NCBI CDD database and SMART protein database showed all 11 trichodiene synthases contain a TRI5 domain (unpublished data).

**TABLE 3:** Gene family analysis for 18 Fusarium species

Species^*a*^	Fusarium species complex^*a*^	Genome size (Mb)	Gene no.^*b*^	Gene family no.	Clustered gene no.^*c*^	Common gene no.^*d*^	Unique gene no.^*e*^	
							Clustered	Nonclustered
F. illudens NRRL 22090	FSSC clade 1	40.3	13,362	8,778	12,459	12,441	18	903
F. solani JS-169	FSSC clade 3 (FSSC 5)	45.2	14,264	9,243	13,778	13,774	4	486
F. virguliforme NRRL 31041	FSSC clade 2	45.4	15,001	9,620	14,297	14,246	51	704
F. protoensiforme NRRL 22178	FSSC clade 3 (FSSC 32)	45.5	16,139	9,659	15,456	15,452	4	683
F. kuroshium UCR3666	FSSC clade 3	46.6	16,485	10,272	16,134	16,130	4	351
F. metavorans NRRL 43489	FSSC clade 3 (FSSC 6)	46.9	16,124	9,650	15,810	15,798	12	314
F. floridanum NRRL 62606	FSSC clade 3	47.4	16,762	10,280	16,257	16,249	8	505
F. falciforme NRRL 43529	FSSC clade 3 (FSSC 3 + 4)	48.2	16,970	9,787	16,035	16,016	19	935
F. euwallaceae HFEW-16-IV-019	FSSC clade 3	48.3	16,501	10,230	16,402	16,402	0	99
F. tuaranense NRRL 46518	FSSC clade 3	48.9	17,570	10,204	16,603	16,559	44	967
F. cuneirostrum NRRL 31157	FSSC clade 2	49.0	15,166	8,596	12,520	12,506	14	2,646
F. ambrosium NRRL 20438	FSSC clade 3 (FSSC 19)	49.0	17,262	10,482	16,603	16,597	6	659
F. brasiliense NRRL 31757	FSSC clade 2	49.4	14,073	7,677	11,544	11,538	6	2,529
F. solani-melongenae CRI 24-3	FSSC clade 3	49.6	15,374	9,136	15,159	15,157	2	215
F. vanettenii MPVI 77-13-4	FSSC clade 3 (FSSC 11)	51.3	15,708	9,142	15,328	15,295	33	380
F. neocosmosporiellum NRRL 22166	FSSC clade 3 (FSSC 8)	54.0	17,936	9,907	16,517	16,322	195	1,419
Subtotal*^f^	N/A	N/A	254,697	152,663	240,902	240,482	420	13,795
Sub-average*^f^	N/A	47.8	15,919	9,541	15,056	15,030	26	862
F. fujikuroi IMI 58289	FFSC	43.8	14,813	9,091	13,960	13,936	24	853
F. oxysporum NRRL 32931	FOSC	59.9	27,347	10,063	24,818	22,989	1,829	2,529
Total**^f^	N/A	N/A	296,857	171,817	279,680	277,407	2,273	17,177
Average**^f^	N/A	48.3	16,492	9,545	15,538	15,412	126	954

a As described in Table S2.

b Total number of genes predicted in this study.

c Total number of all genes detected in gene families.

d Total number of common genes shared by 18 species.

e Unique genes in a specific species comprising genes clustered in gene families or genes that did not belong to any family.

f Summation and the average of statistics for 16 FSSC species (*) and for 18 fusaria genomes (**) per column were listed, respectively; NA, not applicable.

### Comparative genome analysis revealed genomic conservation and uniqueness of CRI 24-3 and other FSSC species.

**FIG 6:**
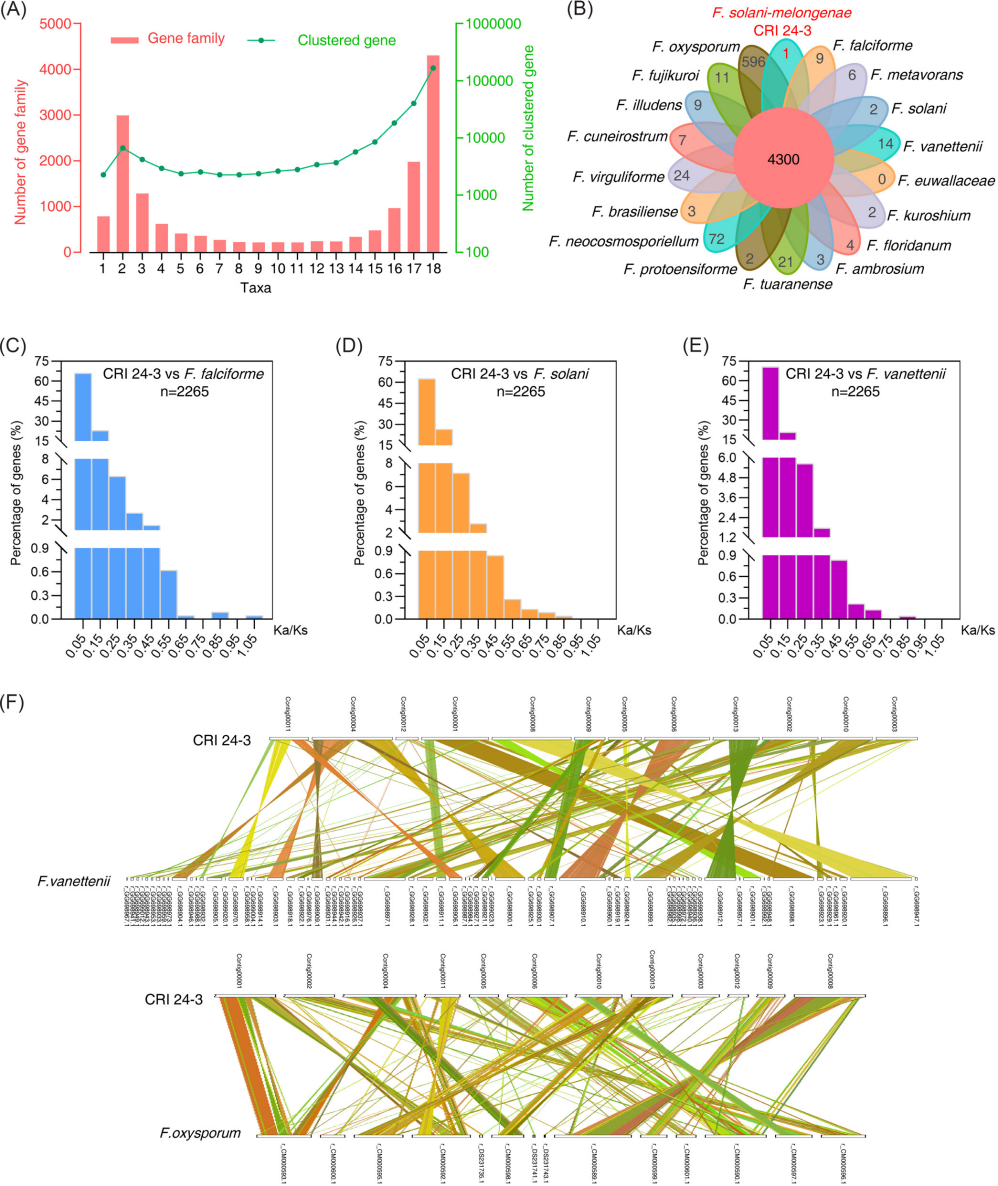
Genome comparison analysis between CRI 24-3 and other Fusarium species. (A) The number of gene families and genes clustered in corresponding families ranged from one to 18. (B) Flower-shaped Venn diagram. The central value indicates the number of all conserved families in 18 Fusarium species; values at the tips indicate the number of unique families preserved in each species. (C to E) K_a_/K_s_ ratio distribution of gene pairs between CRI 24-3 and F. falciforme, F. solani, F. vanettenii. The value following the lowercase letter “n” represents the number of all detected gene pairs. (F) Dual synteny plot showing genome collinearity. Different colored lines were used to connect the orthologous pairs between CRI 24-3 and F. vanettenii (top) or F. oxysporum (bottom).

Genomes from 18 Fusarium species, including 16 sequenced FSSC species, F. fujikuroi, and F. oxysporum were compared to further explore genome diversity and evolution (Table 3; Table S3). A total of 16,130 families (GF1 to GF16,130) containing 279,680 genes were identified and annotated through gene family structure analysis. The results showed that 4,300 essential families were shared by 18 taxa, followed by 2,990 families in two taxa and 786 species-specific families (Fig. 6A and B). Upward trends in the number of genes clustered in 8 to 18 taxa were observed. Approximately 59.6% of the detected genes were grouped in shared families, out of which 1,544 families had single-copy genes. Notably, the effector PKS (EVM0010851.1) mentioned in the previous part was clustered in one shared family (GF2338) with a characteristic acyl transferase domain in Pfam annotation.

Further analysis was performed to explore gene family characteristics. A total of 3,900 genes in 188 common families were unique to 16 FSSC species compared with F. fujikuroi and F. oxysporum (Table S7), implying that these genes evolved after the shared ancestral gene in Fusarium. GF4180, GF1059, and GF3983 were the three largest unique families. Genome size and organization were significantly different among the 18 fusaria. GC content in FSSC species was higher than that in non-FSSC species (Table S3). CRI 24-3 had the third-largest genome among the sequenced FSSC species, following F. vanettenii and F. neocosmosporiellum (Table S3). The number of detected families and subordinate genes in CRI 24-3 was close to the average in FSSC species. A total of 15,157 putative coding genes in the CRI 24-3 genome were shared among 15 other Fusarium species. Notably, 217 unique genes in CRI 24-3 (2 genes comprising one unique family and 215 absent in any of the families) were analyzed using multiple functional databases (Fig. 6B; Table S8). Functional analysis showed that six unique genes encode an ankyrin repeats-containing protein, which is involved in host-microbe interactions (30) and, thus, may be gene-editing targets for future analysis. Most genes only present in CRI 24-3 were significantly enriched in catalytic activities and metabolic processes, such as carbohydrate or secondary metabolite biosynthesis, transport, and metabolism (Fig. S3, S4, and S6).

**FIG 7:**
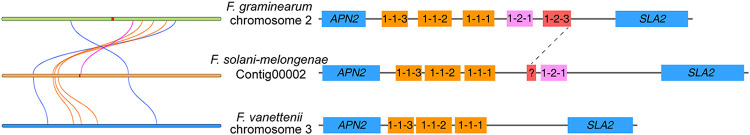
Comparison of *MAT* locus flanked by *APN2* and *SLA2*. A simplified dual synteny plot (left) and genes organization (right) showing orthologous genes pair between F. solani-melongenae and F. graminearum, F. vanettenii. Blue curved lines: *APN2*/*SLA2* genes; orange curved lines: genes (*MAT1-1-1*, *MAT1-1-2*, and *MAT1-1-3*) in *MAT1-1* idiomorph; pink curved line and red rectangles: genes (*MAT1-2-1*, *MAT1-2-3*) in *MAT1-2* idiomorph; question mark: a segment without any characterized domain located in the upstream region of *MAT1-2-1* in F. solani-melongenae shared sequence similarity with *MAT1-2-3* in F. graminearum.

The genomes of three other closely related FSSC species, F. falciforme, F. solani, and F. vanettenii, were used as reference genomes to explore the selection pressure on CRI 24-3. The ratio of nonsynonymous substitution rate (K_a_) to synonymous substitution rate (K_s_) for 2265, 2272, and 2272 ortholog pairs between CRI 24-3 and F. falciforme, F. solani, and F. vanettenii, respectively, was separately determined (Fig. 6C to E). The K_a_/K_s_ values were less than one for the overall analysis, indicating that most orthologs in CRI 24-3 appeared to have undergone purifying selection and were highly conserved. However, a comparison of the CRI 24-3 and F. falciforme genomes identified a K_a_/K_s_ ratio of one fast-evolving gene pair (EVM0014458.1 versus GE10416_g) that was greater than one, indicating positive selection may have occurred during evolution. The genomic collinearity analysis showed that several chromosomal segments in F. vanettenii and F. oxysporum genomes were condensed into one segment in CRI 24-3 with some inversions (Fig. 6F). In addition, some orthologs in CRI 24-3 had many-to-one relationships. At least two query genes in CRI 24-3 matched the same ortholog in the reference species. Notably, significant genomic regions of CRI 24-3 could not be collinearly coupled with those in the F. oxysporum genome, supported by a 35.9% collinearity rate (the ratio of colinear ortholog pairs to all ortholog pairs), which was less than that between CRI 24-3 and F. vanettenii (77.6%). The result indicated that CRI 24-3 is phylogenetically distant from F. oxysporum. *MAT* locus is critical for producing ascospores in Fusarium species. Herein, structural organizations of mating-type (*MAT*) locus in F. solani-melongenae CRI 24-3, F. graminearum PH-1, and F. vanettenii 77-13-4 were compared based on a simplified collinearity analysis (Fig. 7). In contrast to F. vanettenii 77-13-4 only bearing *MAT1-1* genes, CRI 24-3 contained *MAT1-1* and *MAT1-2* idiomorph genes closely linked at a single *MAT* locus as F. graminearum, except that CRI 24-3 is a deficiency of *MAT1-2-3*. Only a segment without any characterized functional domain located in the upstream region of *MAT1-2-1* in CRI 24-3 shared sequence similarity with *MAT1-2-3* in F. graminearum.

## DISCUSSION

In the present study, the causal agent of FRST, F. solani-melongenae isolate CRI 24-3, was isolated and subjected to whole-genome sequencing. Typical symptoms of sweetpotatoes with FRST include surface and sunken rot in the storage root, and stem decay with red perithecia, which are different from those of Fusarium root rot, Fusarium adventitious root rot, and Fusarium root rot and stem canker (31–33). F. solani was reported as the pathogen of these diseases. However, the causal agent of Fusarium adventitious root rot has not been fully elucidated because Hu and Zhou (25) identified F. solani-melongenae as the major pathogen. Booth (6) classified F. solani-melongenae into a *formae speciales* of F. solani, whereas Ye (34) reported that F. solani-melongenae was a variant of F. solani. Findings of the present study showed F. solani-melongenae and F. solani are phylogenetically distinct species as shown by analyses of conidia morphology, molecular phylogenetics, and multiple genome comparisons. A high-quality genome sequence of CRI 24-3 with a relatively high GC content was obtained by combining ONT long-read sequencing with Illumina short-read error correction. Whereas GC content is a taxonomy indicator and positively correlated with genome size in bacteria (35, 36), GC content was negatively correlated with genome size in the FSSC, consistent with results on monocots and geophytes (37, 38). This can be attributed to balancing between GC content and genome size because more energy is required to synthesize more GC bases in larger genomes.

Average nucleotide identity (ANI) is widely used in determining genomic similarities in prokaryotes (39). However, it is challenging to assess fungal phylogenetic relationships using the ANI method because fungal genomes are larger and more complicated than those of prokaryotic. A phylogenomic analysis is an ideal alternative. F. falciforme was the closest relative of CRI 24-3 among 15 other sequenced fusaria in the phylogenomic tree, and it is also a plant pathogen causing root and stem wilt (40, 41). Moreover, further studies should be conducted to explore phylogenetic relationships among all FSSC species by including genomes of the other species that are not currently sequenced. Notably, the selected data set and construction methods can affect the topological structure of the phylogram (42, 43). In the current study, the ITS tree using ML and NJ methods consistently identified CRI 24-3 as F. solani-melongenae (unpublished data). However, the ITS sequence is short and less informative than a multiple-locus-based phylogenetic analysis. This was well illustrated in the three-loci tree reported here.

CAZymes and secondary metabolite synthases play essential roles in primary and secondary metabolic pathways, respectively, and some of these enzymes promote pathogen virulence (14, 18, 44). GH was the largest CAZymes class in CRI 24-3 similar to several other Fusarium species (12, 20, 45), indicating that GH plays a significant role in Fusarium. Endo-β-1,4-xylanases (EC 3.2.1.8) from GH11 are key enzymes that hydrolyze β-1,4 glycosidic bonds between two d-xylopyranosyl residues of xylan polymers (a major component of the plant cell wall), whereas the xylanolytic function of three hypothetical GH11s in CRI 24-3 proteome remains to be explored. Fusarium produces abundant secondary metabolites, including polyketide, nonribosomal peptide, and trichothecene, which are toxic and significantly cause diseases (46). However, others are beneficial. For instance, gibberellin from F. fujikuroi is used as a plant growth regulator (47). Genome comparison analyses of Fusarium have identified tandem array arrangements of secondary metabolites synthases, including PKS, NPRS, and TS, which catalyze the synthesis of secondary metabolites as mentioned above. The number, type, and distribution of these synthases exhibit significant diversity among Fusarium species, even in species sharing a common ancestor (13, 17). For instance, 16 PKSs, 19 NRPSs, and 8 TSs were identified in the F. graminearum proteome, whereas 12 PKSs and 12 NRPSs without a TS were detected in the F. vanetteii 77-13-4 proteome. TRI5 and other TSs are the key synthase cluster that involves in the biosynthesis of trichothecene mycotoxins, which have not been previously identified in FSSC species (12). However, the findings in the present study showed that CRI 24-3 genome encodes two trichodiene synthases (named TRI5-1 and TRI5-2) and several other TSs. Interestingly, TRI5-1 and TRI5-2 shared higher similarities with an uncharacterized protein (XP_003047531.1) of F. vanettenii and other homologous trichodiene synthases in the divergent genus, whereas they were distantly related to those of Fusarium species. Further studies should explore whether these unique synthases produce trichothecene mycotoxins and promote the pathogenicity of CRI 24-3. Studies on host-pathogen interactions are used to explore pathogenic mechanisms and the findings provide a basis for the development of strategies to control diseases. A total of 216 putative effector genes were identified in the CRI 24-3 genome. Host-plant resistance genes corresponding to these effector genes can be detected rapidly using bioinformatics tools such as PRGdb or a yeast hybridization assay based on the “gene-for-gene” hypothesis (48). These resistant genes can be used to develop resistant cultivars through hybridization or transgenic breeding.

Comparative genome analysis enhances the understanding of evolutionary relationships and gene function among multiple species through the identification of gene sequences and their order, classification of gene families, and assessment of genome collinearity. Gene family analysis in the current study revealed that more than half of the predicted genes in 18 Fusarium species were clustered in a small proportion of the detected gene families, indicating that these genes may undergo horizontal gene transfer, which is common among pathogens (18, 49), and the species may adopt similar survival strategies and pathogenicity mechanisms. High-quality genome assembly and accurate gene prediction are necessary for determining collinearity relationships between divergent species. Although whole-genome phylogenetic analysis showed that F. falciforme was closely related to CRI 24-3, it only had a 37.5% collinearity rate (11,100 colinear genes/30,375 detected genes) with CRI 24-3, possibly due to the incomplete assembly with too many gaps in the F. falciforme genome. The many-to-one relationships between CRI 24-3 and other Fusarium species can be attributed to genome duplication. Therefore, the resulting potential functional redundancy or changes in related traits should be explored further. *MAT* locus component and organization in CRI 24-3 is divergent from that in heterothallic F. vanettenii and homothallic F. graminearum. F. neocosmosporiellum is also a homothallic FSSC species harboring *MAT1-1* and *MAT1-2* genes (50) as F. solani-melongenae. However, the *MAT* locus and the organization of the flanking genes in F. neocosmosporiellum differs from that in F. solani-melongenae, suggesting that they may evolve independently within the FSSC. To date, it has not reached a definite conclusion about whether homothallic species evolved from heterothallic species in Fusarium; by contrast, previous studies in *Cochliobolus* and *Neurospora* species supported the idea that homothallic species evolved from heterothallic species (51–53).

In summary, a novel Fusarium disease causing root and stem rot in sweetpotatoes was identified in the present study. The chromosome-level draft genome of the causal agent, F. solani-melongenae isolate CRI 24-3, was sequenced for the first time. The results will guide the assembly of other closely related fusarium genome sequences. Comprehensive genomic comparative analysis of the genomes of the sequenced FSSC members can improve understanding of the genetic characteristics of the FSSC and even in the entire genus. Moreover, further analysis of the virulence factors that promote infection in plants will provide a basis for the development of Fusarium disease management strategies, thus minimizing economic losses.

## MATERIALS AND METHODS

### Fungal isolation, pathogenicity test, and morphological analysis.

FRST-infected sweetpotatoes were evaluated, and photographs were taken (EOS 60D, Canon) at the experimental station of the Crops Research Institute, Guangdong Academy of Agricultural Sciences, Guangzhou, Guangdong, China. Newly infected stems were collected for fungal isolation in the laboratory. The infected stems were rinsed with clean water and then cut into small sections using a sterilized blade in a laminar flow cabinet. The sections were prepared as follows. They were (i) soaked in 75% (wt/vol) alcohol for 30 s and 0.1% (wt/vol) mercury dichloride for three min, (ii) washed thrice using sterilized water and dried on sterilized filter paper, and (iii) placed on PDA plates, and cultured in an incubator (25°C, 12 h photoperiod, and 75% relative humidity). Mycelial discs (0.5 cm diameter) from fungi boundaries were obtained using a sterilized puncher and transferred to new PDA plates for pure culture. CRI 24-3 was the most virulent fungal isolate, therefore, it was selected for subsequent analyses.

For the pathogenicity test, small pieces of healthy sweetpotato storage root (2 to 3 cm thickness) were sterilized, as described above. Mycelial discs (1 cm diameter) of CRI 24-3 were then inoculated onto the center of each sample using a sterilized puncher. The negative-control was only inoculated with an agar disc of the same size. All the treatments were moistened using sterilize water and incubated for three to five days at 25°C with a 12 h photoperiod. Each treatment comprised three biological replicates.

CRI 24-3 was cultured on PSA and SNA medium for four days to evaluate its growth rate. The average colony diameter of the three replicates was calculated after culturing. An optical microscope (Axio Lab.A1, Carl Zeiss) was used to document the morphology of CRI 24-3, and conidia and ascospore size were determined using ZEISS ZEN software version 3.4 (*n* = 12).

### Genome sequencing, *de novo* assembly, and annotation.

High-quality genomic DNA was extracted from CRI 24-3 and used for Oxford Nanopore PromethION plus 125 bp paired-end Illumina Hiseq sequencing, following previously described procedures (54). Adapters and low-quality/short reads less than 2 kb were deleted, and raw data in fastq format were used to generate contigs of the preliminary genome using the Flye *de novo* assembler (55). A more accurate draft genome was assembled through long error reads correction and short reads were polished using Racon v1.3.1, LoRDEC v0.5 with modified parameters (LoRDEC-0.5 -k 21 -T 4 -s 3), and Pilon v1.22 (56–58). For quality assessment, the Illumina HiSeq clean reads were aligned to the genome assembly using BWA v0.7.10 (59), and the resulting alignments were analyzed using SAMTools commands. BUSCO v5.2.2 (60) was then used to assess the completeness of the genome assembly using the lineage data sets of sordariomycetes_odb10. Circos v0.66 (61) was used to describe the genomic structure of CRI 24-3.

Prediction of CRI 24-3 genes was integrated using three complementary gene-searching methods (*Ab initio*, homolog based, and RNA-seq based) as reported by Jiang et al. (62). BLAST v2.2.29 (63) was used to align sequences of predicted genes/genes products against the Nr database. GO analysis was performed using Blast2GO v2.5 (64) based on Nr hits. Pfam annotation was conducted using the Pfam database through the HMMER v3.0 webserver (65). Furthermore, KOG and KEGG functional enrichment analysis was performed. Specialized functional annotations were assigned to protein-coding genes by evaluating amino-acid sequence similarity between the query conserved domains and proteins in the CAZy v4.0 (66) and PHI v4.8 databases (67) using HMMER v3.0 (65). Amino-acid sequences of three β-1,4-xylanases in CRI 24-3 (i.e., FsmGH11.1, FsmGH11.2, and FsmGH11.3) were aligned with the NhGH11 sequence (GenBank accession no. XP_003050975.1) using CLUSTALW (https://www.genome.jp/tools-bin/clustalw) and the multiple sequence alignment was inspected using ENDscript 3.0 (https://espript.ibcp.fr/ESPript/ESPript/index.php).

### Molecular phylogenetic analysis.

*ITS, RPB2*, and *TEF 1-α* sequences were extracted from the CRI 24-3 genome assembly. The 51 other sequences of each locus from 34 FSSC phylogenetic species were retrieved from the NCBI database for 3-gene tree construction (Table S2). MUSCLE 3.8.31 was used for multiple nucleotide sequence alignment and poor alignments were edited using Gblocks 0.91b (68) with modified parameters (t = DNA -b4 = 5 -b5 = a). The ML tree based on the concatenated *ITS, RPB2*, and *TEF 1-α* sequences was constructed using PhyML 20151210 (69) with 1000 bootstrap replicates. Similar parameters were used for the construction of the phylogenomic tree based on all single-copy orthologous genes from 16 FSSC species, including CRI 24-3 (Table S3). The method for selecting a single-copy gene is shown in the next section.

Before protein phylogenetic analysis, TRI5-1 and TRI5-2 from CRI 24-3 used as protein queries were compared to the NCBI Nr database through a BLASTP search. Most of the top 100 hits corresponded to the recognized TRI5/trichodiene synthase/terpenoid synthase. Nine orthologs with definite functional annotation, over 65% sequence similarity, and 84% coverage from the Fusarium and other genera were selected for phylogenetic relationship analysis along with TRI5-1 and TRI5-2, which was inferred by using ML with 1000 bootstrap replicates conducted in MEGA7 (70).

### Comparative genome analysis.

Genome sequences of 15 FSSC species along with F. fujikuroi and F. oxysporum (Table S3) were obtained from the NCBI database. Eleven Fusarium genomes initially annotated in GenBank format were reannotated using methods as described in Genome sequencing, *de novo* assembly, and annotation. OrthoMCL (71) was then used to determine the common families shared by all 18 species and the unique families in individual species based on gene family colinear relationships. The Pfam database was used to annotate all gene families, and unique genes in CRI 24-3 were annotated using the GO, KOG, and KEGG databases. Single-copy homologous genes shared by all species were then extracted for further analyses. K_a_/K_s_ rate for single-copy orthologs between CRI 24-3 and F. falciforme, F. solani, and F. vanettenii were evaluated using the PAML package. Genes with: (i) K_a_/K_s_ rate ratio <1 were considered to have undergone purifying selection, (ii) K_a_/K_s_ rate ratio =1 were considered to have undergone neutral selection, and (iii) K_a_/K_s_ rate ratio >1 were considered to have evolved under positive selection. Sequences and positions of protein-coding orthologs in the CRI 24-3 genome were compared with those of F. vanettenii and F. oxysporum using BLAST for genome-wide collinearity analysis. The genomic and gene colinear relationship were analyzed using MCScanX (72) and TBtools (73), respectively.

### Data availability.

The genome sequences and annotation of F. solani-melongenae isolate CRI 24-3 were deposited in the NCBI database (GenBank accession no. GCA_023101225.1). The other relevant data supporting the findings in the current study are presented in this paper or supplemental material.
