# Supplementary material for: Whole-Genome Sequencing and Comparative Genome Analysis of Fusarium solani-melongenae Causing Fusarium Root and Stem Rot in Sweetpotatoes
Source: Microbiol Spectr. 2022 Jul 7;10(4):e00683-22. doi: 10.1128/spectrum.00683-22 (PMC9430127; doi:10.1128/spectrum.00683-22)
Supplement: Supplemental file 1 — Supplemental material. Download spectrum.00683-22-s0001.pdf, PDF file, 1.8 MB [file spectrum.00683-22-s0001.pdf]

Supplemental Tables and their Legends

TABLE S1 Length distribution of clean reads

TABLE S2 GenBank accession numbers of Fusarium sequences used in Figure 4a

[a]Seven heterothallic mating populations (MPs) of *N. haematococca*.

[b]ACCC: Agricultural Culture Collection of China; CBS: Westerdijk Fungal Biodiversity Institute; NRRL: Agricultural Research Service Culture Collection; VG: Working collection of J. Van Niekerk; CPC: Collection of P.W. Crous; UCR: University of California, Riverside; UFMG-CM F: Collection of Microorganisms, DNA and Cells of Minas Gerais Federal University.

[c]FSSC: *Fusarium solani* species complex.

TABLE S3 Strain data for 18 *Fusarium* species used in comparative genomic analysis

[a,b]As described in **TABLE S2**.

[c]FSSC: *Fusarium solani* species complex; FFSC: *Fusarium fujikuroi* species complex, FOSC: *Fusarium oxysporum* species complex.

TABLE S4 CAZymes detected in 24-3 proteome

[a]Proteins with more than one CAZyme domain are indicated in red font, while domains with the lowest E-value s are indicated using bold font. Endo-β-1,4-xylanases within GH class are indicated in blue font. Proteins belonging to more than one family are indicated in yellow.

[b]CAZyme family that query protein belonged to.

[c]The initiation and termination sites of CAZyme domain in the query protein.

[d]Length of CAZyme that query protein matched.

[e]Initiation and termination sites of CAZyme domain that query protein matched.

TABLE S5 PHI proteins of CRI 24-3

[a]Accession number of reference sequence in PHI database.

[b]Ratio of identical amino acid residues to all residues for alignments between the query and the reference in PHI database.

[c]Expectation value of alignments between query and reference in PHI database.

[d]Name and GenBank accession number of gene that query protein coding gene matched.

[e]Systematic name and PHI ID number of the pathogenic species.

[f]Defined phenotype of transgenic strain.

TABLE S6 Effectors predicted in CRI 24-3

[a]A total of 36 effectors predicted using PHI database are indicated in yellow, while the other 180 effectors predicted using EffectorP v2.0 are indicated in blue.

[b]CAZyme family that query protein belonged to according to **TABLE S4**.

[c]Defined phenotype of transgenic strain according to **TABLE S5**.

TABLE S7 Analysis of the top ten unique gene families in 16 species in the FSSC

[a]Gene families in 16 FSSC members but not in *F. oxysporum* and *F. fujikuroi*.

[b]Gene IDs and corresponding species names.

TABLE S8 Functional annotations of 217 unique genes in CRI 24-3

[a]Unique genes only found in CRI 24-3.

[b]One gene family, including 2 unique genes, is listed.

Supplemental Figures/Tables and their corresponding legends are provided in the Supplemental Material file.

Supplemental Figures and their legends

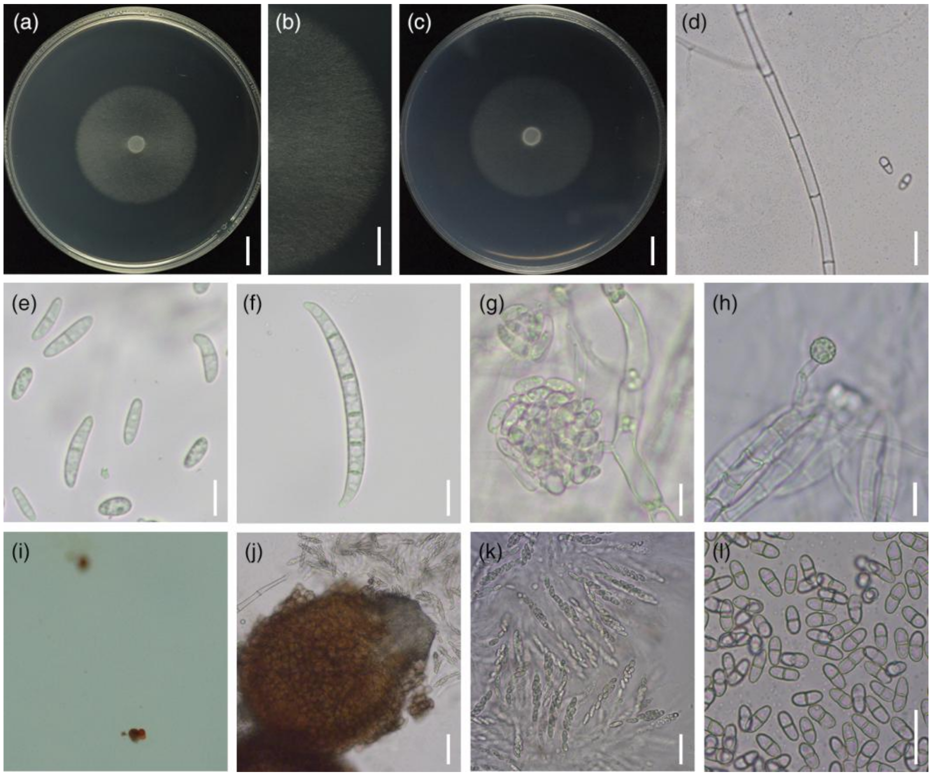

FIG S1 Morphological characteristics of CRI 24-3 on SNA. (a-c) Colony from the surface and reverse after culture for four days. Bar = 1 cm. (d) Aerial hyphae. (e) Microconidia. (f) Macroconidia. (g) Conidia in false head. (h) Chlamydospore. Bar = 10 μm. (i) Perithecia. (j) Enlarged perithecium. Bar = 50 μm. (k) Ascus bearing (l) Eight ascospores. Bar = 20 μm.

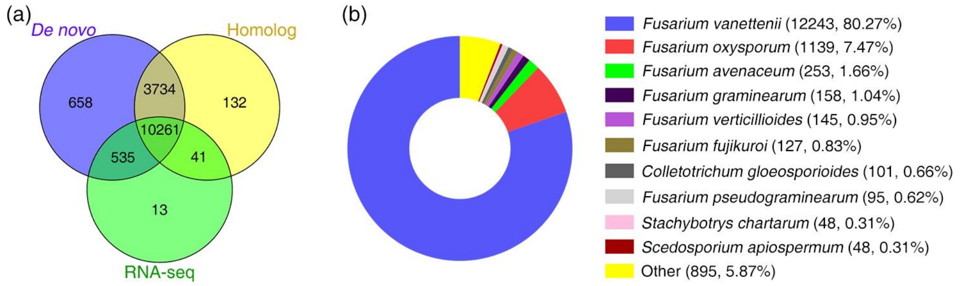

FIG S2 Predictions and genome-wide alignments of CRI 24-3 genome. (a) Venn diagram showing the number of genes in CRI 24-3 genome predicted by three methods. A total of 15,374 putative genes were predicted. (b) Homologus protein distribution based on Nr database. Matched species, the number of homologs and its ratio to all 15,252 Nr annotation proteins are shown.

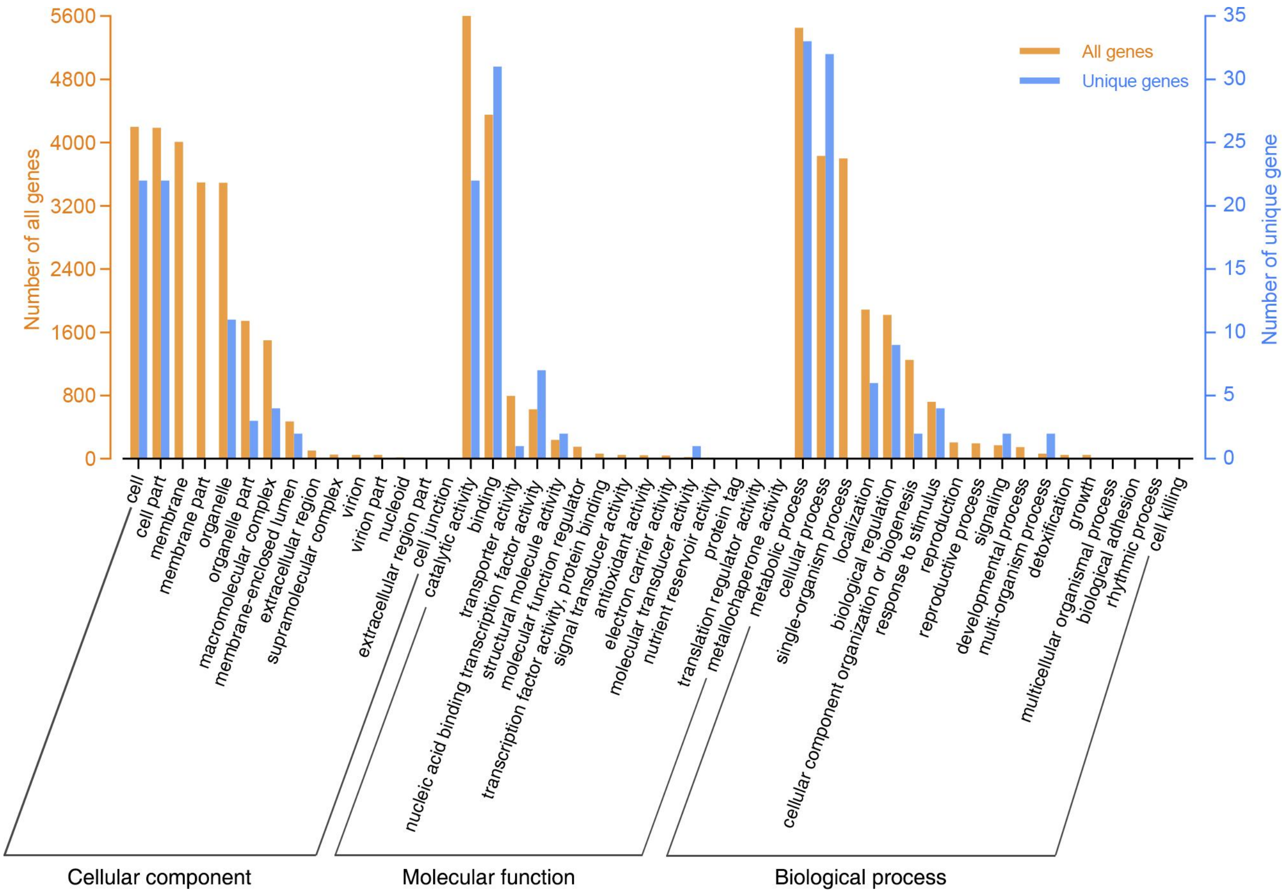

FIG S3 GO terms of CRI 24-3. A total of 10,655 genes (orange) and 61 unique genes (blue) were functionally interpreted.

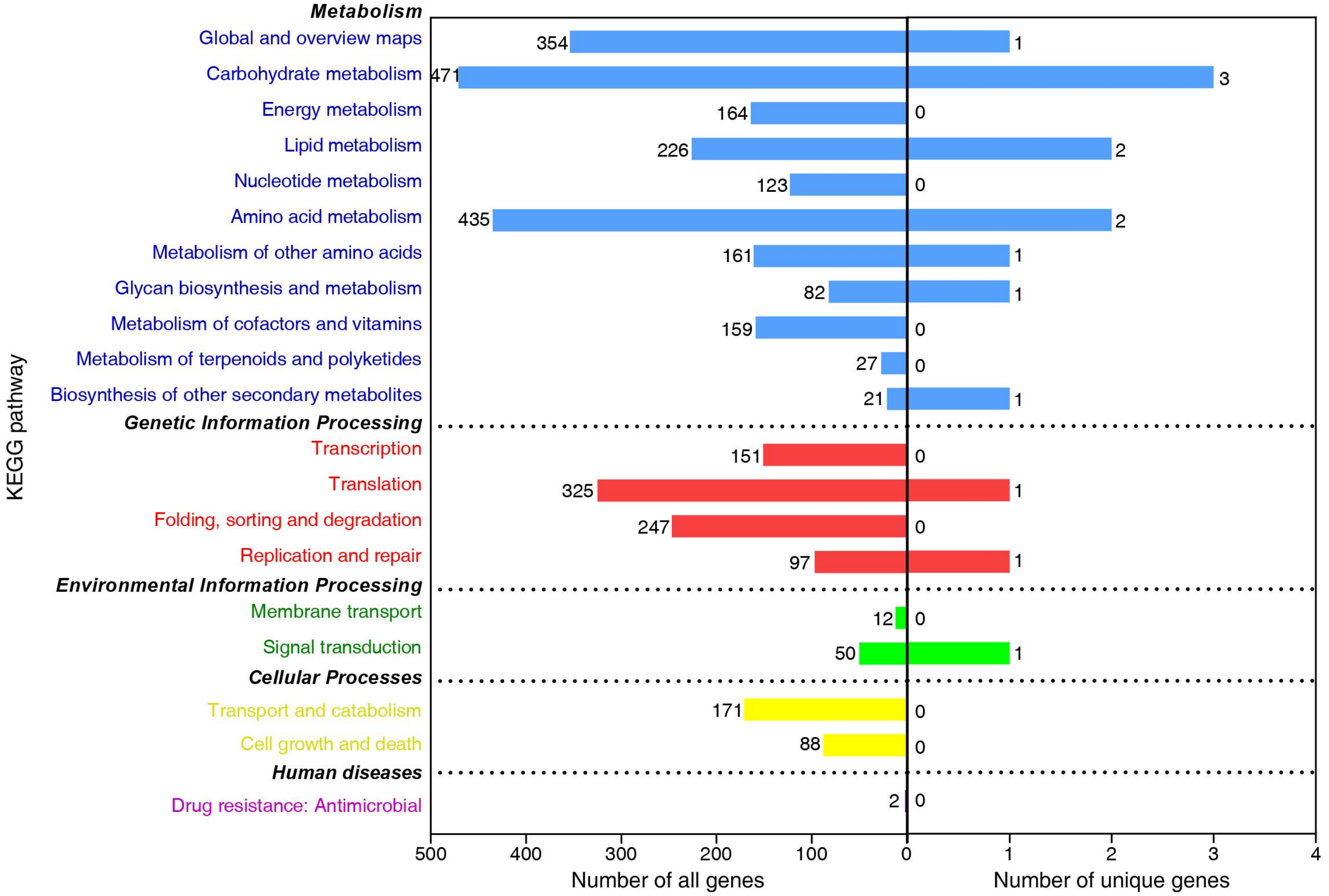

FIG S4 KEGG classification analysis of CRI 24-3. All 2,329 genes and 7 unique genes were classified into five major categories.

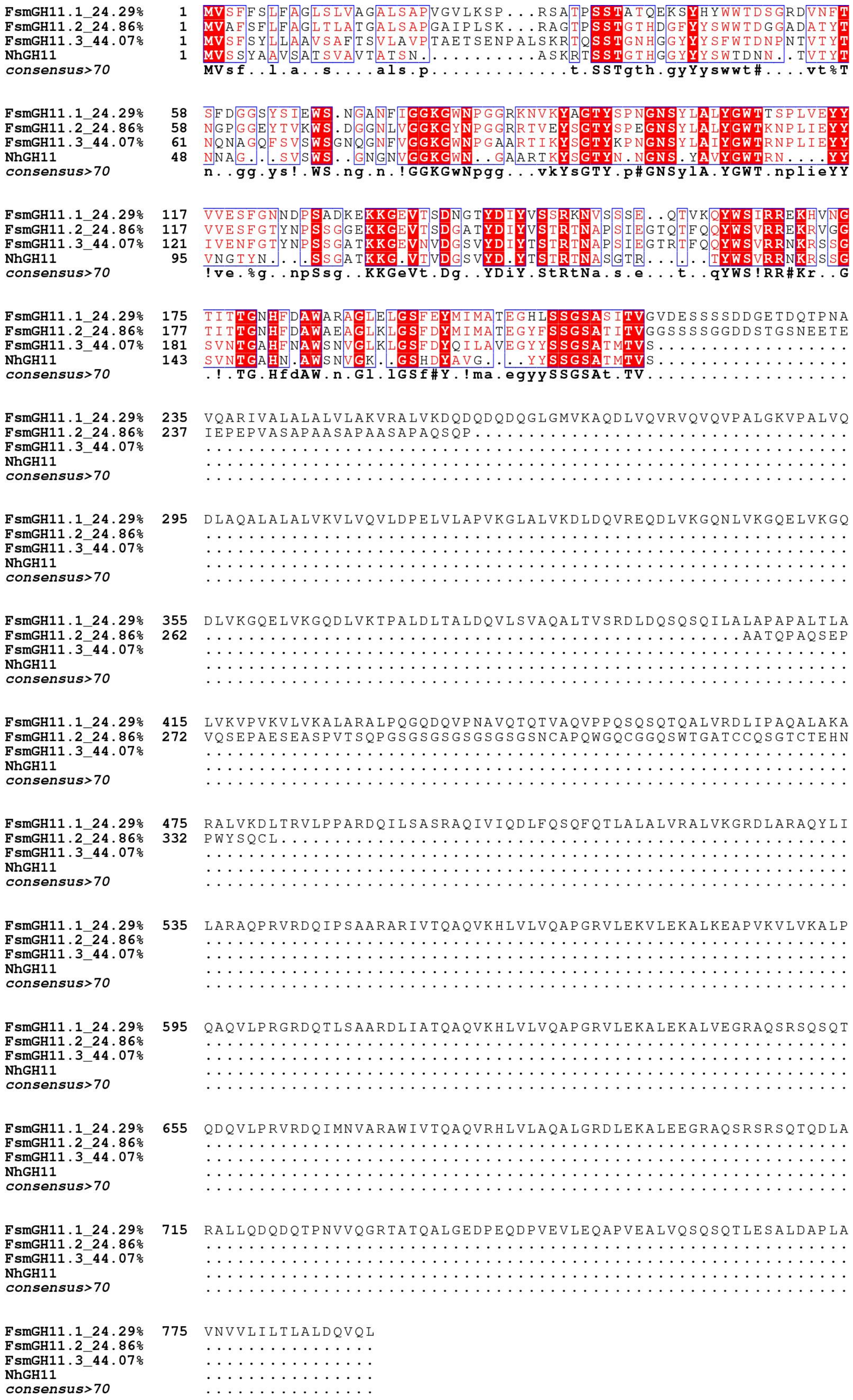

FIG S5 Amino acid sequence alignment of FsmGH11.1 (EVM0007517.1), FsmGH11.2 (EVM0010092.1), FsmGH11.3 (EVM0014019.1), and NhGH11 (XP_003050975.1).

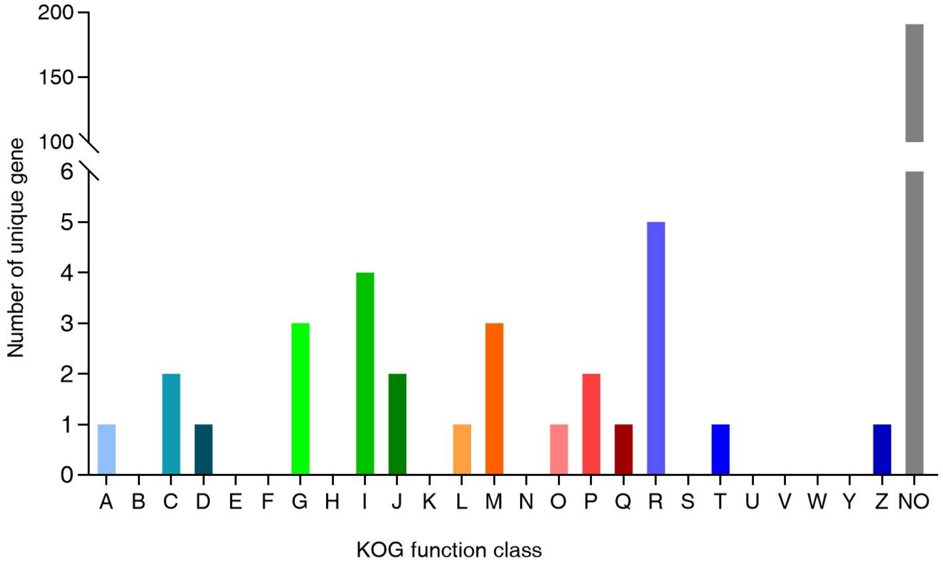

A: RNA processing and modification (1)
B: Chromatin structure and dynamics (0)
C: Energy production and conversion (2)
D: Cell cycle control, cell division, chromosome partitioning (1)
E: Amino acid transport and metabolism (0)
F: Nucleotide transport and metabolism (0)
G: Carbohydrate transport and metabolism (3)
H: Coenzyme transport and metabolism (0)
I: Lipid transport and metabolism (4)
J: Translation, ribosomal structure and biogenesis (2)
K: Transcription (0)
L: Replication, recombination and repair (1)
M: Cell wall/membrane/envelope biogenesis (3)
N: Cell motility (0)
O: Posttranslational modification, protein turnover, chaperones (1)
P: Inorganic ion transport and metabolism (2)
Q: Secondary metabolites biosynthesis, transport and catabolism (1)
R: General function prediction only (5)
S: Function unknown (0)
T: Signal transduction mechanisms (1)
U: Intracellular trafficking, secretion, and vesicular transport (0)
V: Defense mechanisms (0)
W: Extracellular structures (0)
Y: Nuclear structure (0)
Z: Cytoskeleton (1)
NO: No KOG annotation (191)

FIG S6 KOG functional analysis of 26 unique genes in CRI 24-3.
